# Supplementary material for: Single‐cell mRNA profiling reveals the hierarchical response of miRNA targets to miRNA induction
Source: Mol Syst Biol. 2018 Aug 27;14(8):e8266. doi: 10.15252/msb.20188266 (PMC6110312; doi:10.15252/msb.20188266)
Supplement: Supplementary file 1 — Appendix [file MSB-14-e8266-s001.docx]

APPENDIX: Supplemental Figures and Tables

**Table of Contents**

**Appendix Figure S1 2**

**Appendix Figure S2 3**

**Appendix Figure S3 4**

**Appendix Figure S4 5**

**Appendix Figure S5 6**

**Appendix Figure S6 7**

**Appendix Figure S7 8**

**Appendix Figure S8 9**

**Appendix Figure S9 10**

**Appendix Figure S10 11**

**Appendix Figure S11 12**

**Appendix Figure S12 13**

**Appendix Table S1 14**

**Appendix Table S2 15**

**References 16**

##

##

##


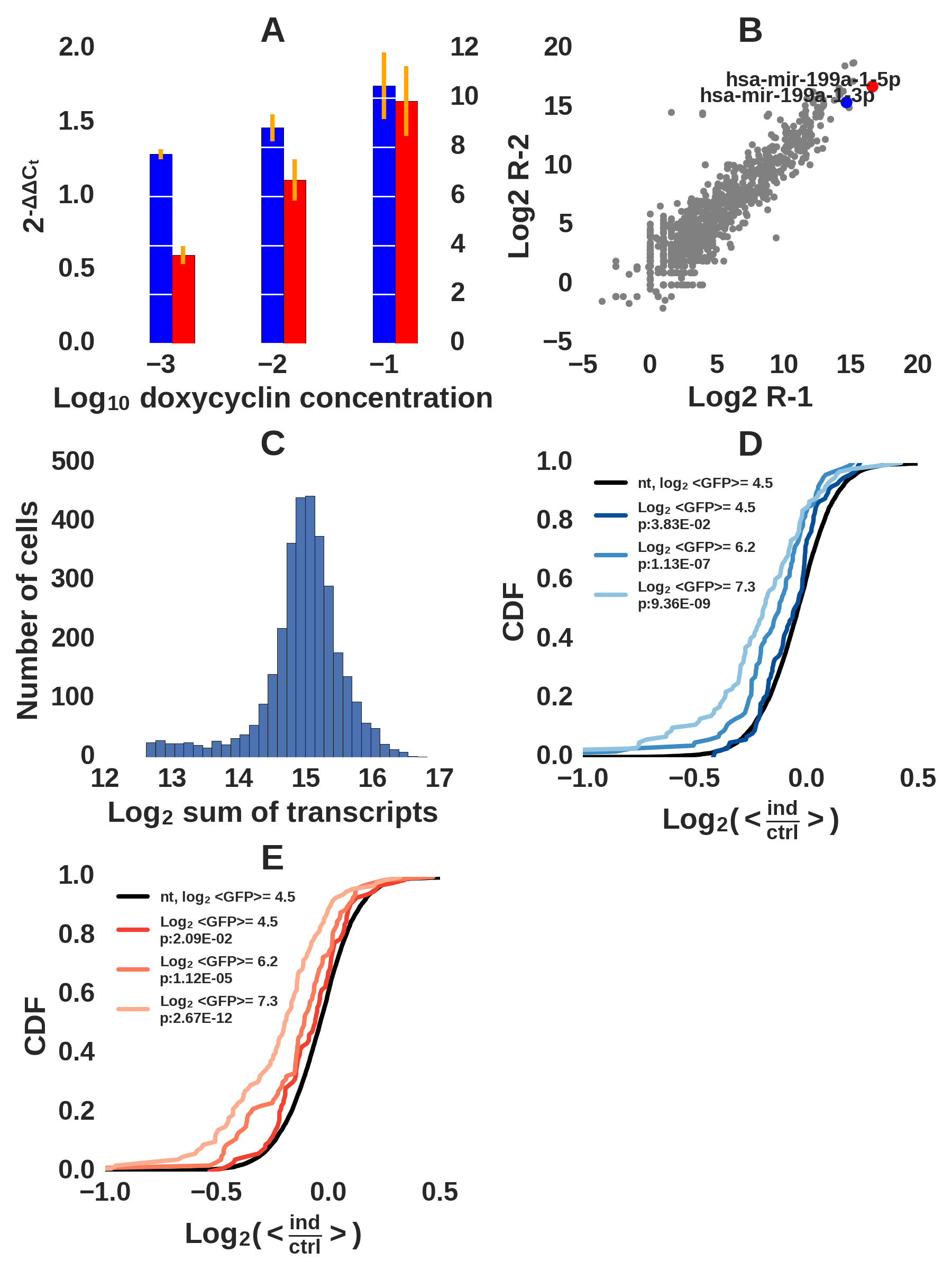


**Appendix Figure S1.** **Characterization of hsa-miR-199a-5p and hsa-miR-199a-3p miRNA activity. A.** Relative hsa-miR-199-3p (blue, left y-axis) and hsa-miR-199-5p (red, right y-axis) miRNA levels in doxycycline-induced cells compared to the non-induced cells, measured by quantitative PCR, demonstrate that the two miRNAs are co-expressed. The C_t_ values obtained for each set were normalized to the levels of hsa-miR-16 and to the values from non-induced cells. Error bars indicate standard deviations from 2 experiments. **B**. Expression of miRNAs in fully induced HEK cells as measured by Clip-Seq. Two replicates are correlated, hsa-miR-199-5p and hsa-miR-199-3p are indicated. **C.** Number of transcripts identified in individual i199 cells. **D,E.** Downregulation of top 100 predicted targets of the miRNAs in i199 cells with different levels of GFP. Three sets of cells (200 cells each) with increasing GFP expression levels were used, showing that the downregulation of hsa-miR-199-3p (blue lines, **D**), and hsa-miR-199-5p (red lines, **E**) targets increases with level of GFP expression; the distribution of log- fold changes of non targets is shown in black. *P* values are from the Kolmogorov-Smirnov test comparing the distributions of targets with that of non-targets.


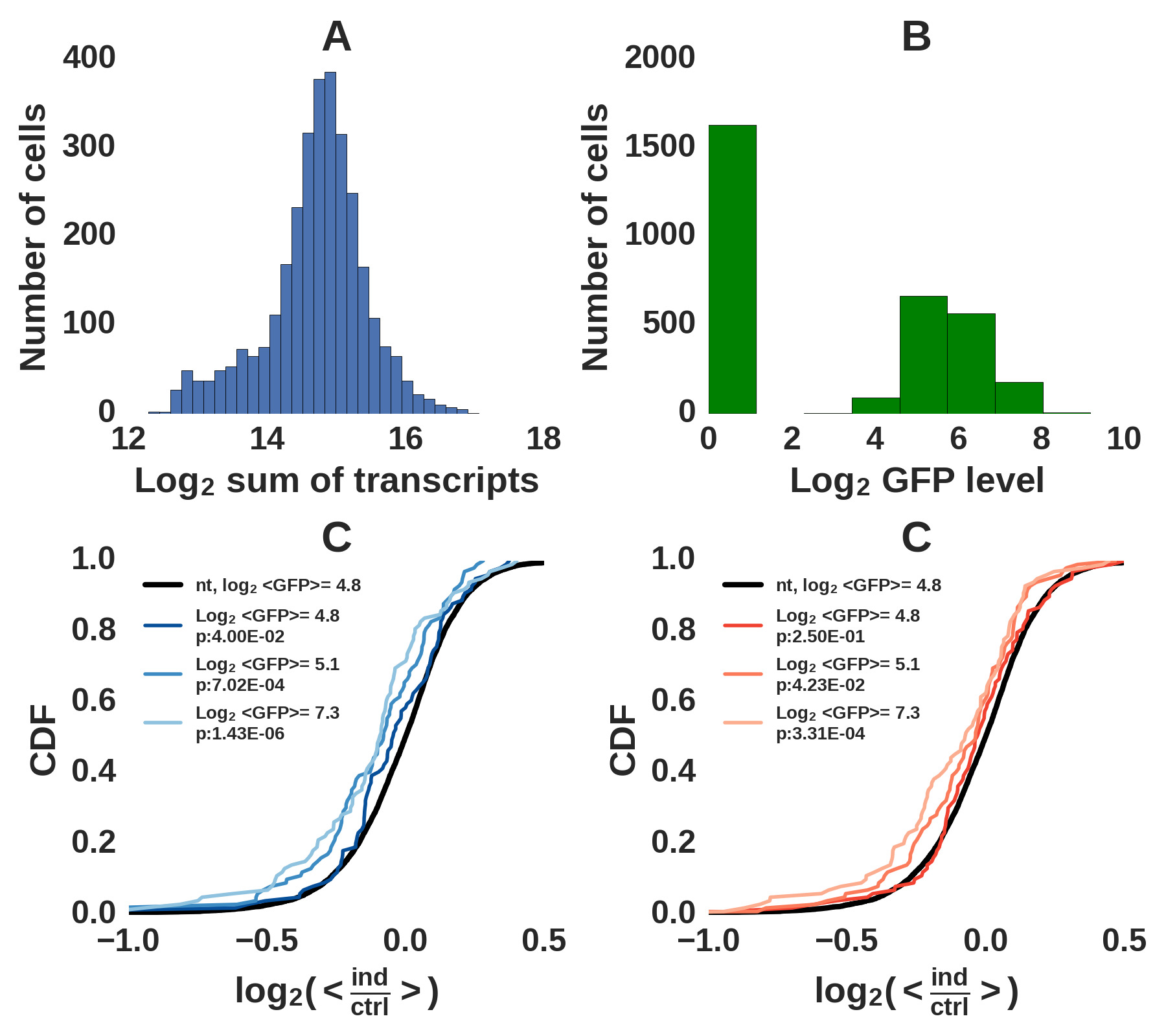


**Appendix Figure S2. Characterization of miRNA activity in single i199-KTN1 HEK cells**. **A.** Count of transcripts identified from each individual gene in single i199-KTN1 cells. **B.** Normalized GFP mRNA expression distribution in single cells. **C,D.** Downregulation of top 100 predicted targets of the miRNAs in i199-KTN1 cells with different levels of GFP. Three sets of cells (200 cells each) with increasing GFP expression levels were used, showing that the downregulation of hsa-miR-199-3p (blue lines, **C**), and hsa-miR-199-5p (red lines, **D**) targets increases with level of GFP expression; the distribution of log-fold changes of non targets is shown in black.


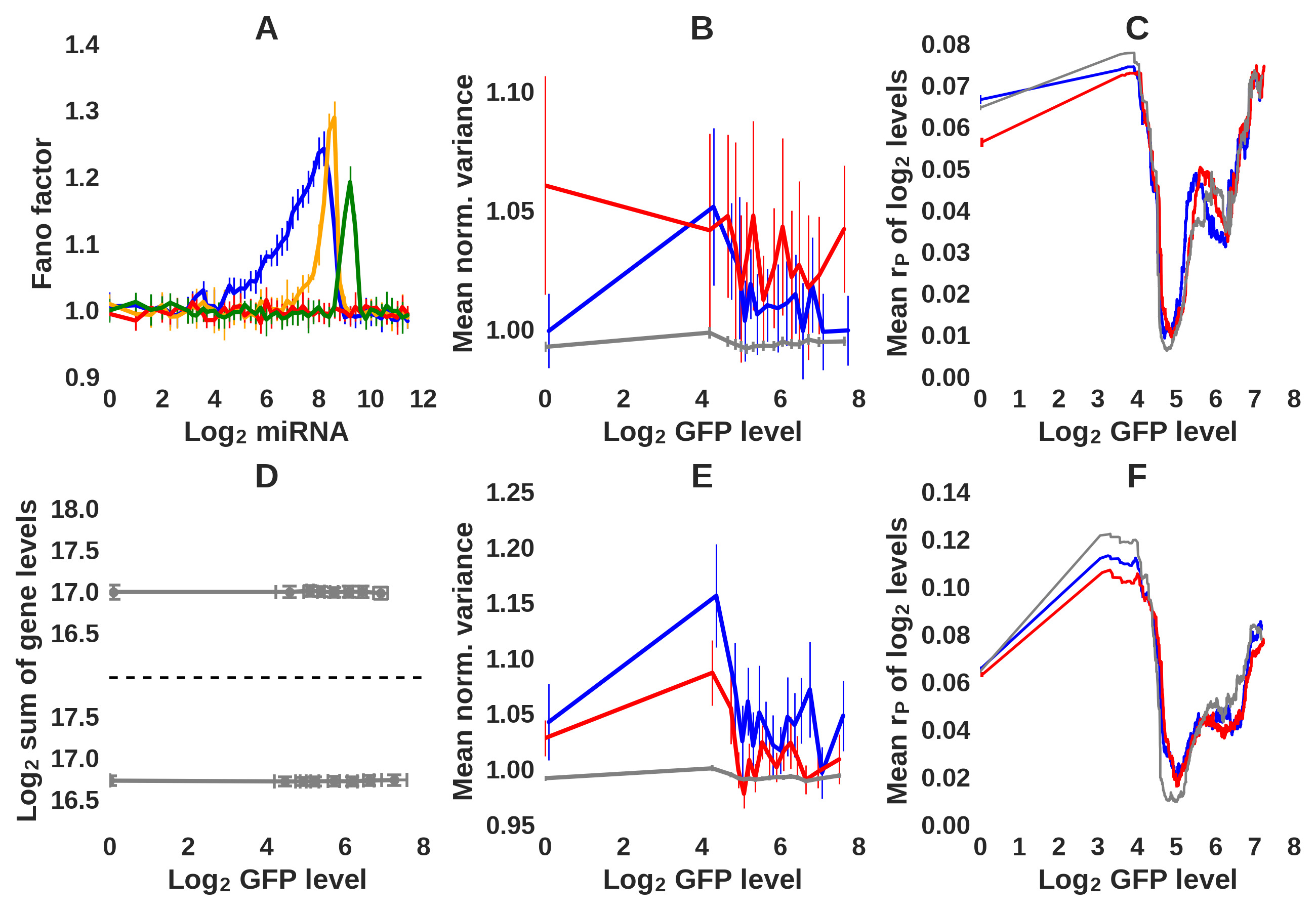


**Appendix Figure S3. Expected and observed response of miRNA targets to miRNA induction in single cells; additional information. A.** Fano factor, of *in silico* target levels across cells, calculated in function of miRNA expression, from the simulation trajectories. The panel corresponds to panel B, Figure 2, where C_V_ is calculated using the same data. **B, E.** Normalized variance (using PAGODA package [(Fan *et al*, 2016)](https://paperpile.com/c/tsJs0v/hUIF)) of 100 lowest $A_{F}^{C}$hsa-miR-199a-5p (red) and hsa-miR-199a-3p (blue) targets and all genes (grey) in the i199 **(B)** and i199-KTN1 **(E)** cells, in function of log_2_ GFP expression in the same cells; see Methods about PAGODA normalization and calculation details. **D.** Total expression (log_2_ sum of TPMs) of 1000 random genes in the i199 **(**lower line**)** and i199-KTN1 **(**upper line**)** cells. **C,F.** Mean Pearson pairwise correlation coefficients for miRNA targets in function of GFP expression in i199 **(C)** and i199-KTN1 **(F)** cells. Mean from 50 calculation evaluations of random selection of 100 non-target genes is shown as grey line. Means were calculated from the two hundred cells with GFP expression closest to a specific expression level (**C,D,F**). For **A,C,D** and **F** panels standard deviations are shown, for **B** and **E** standard error.


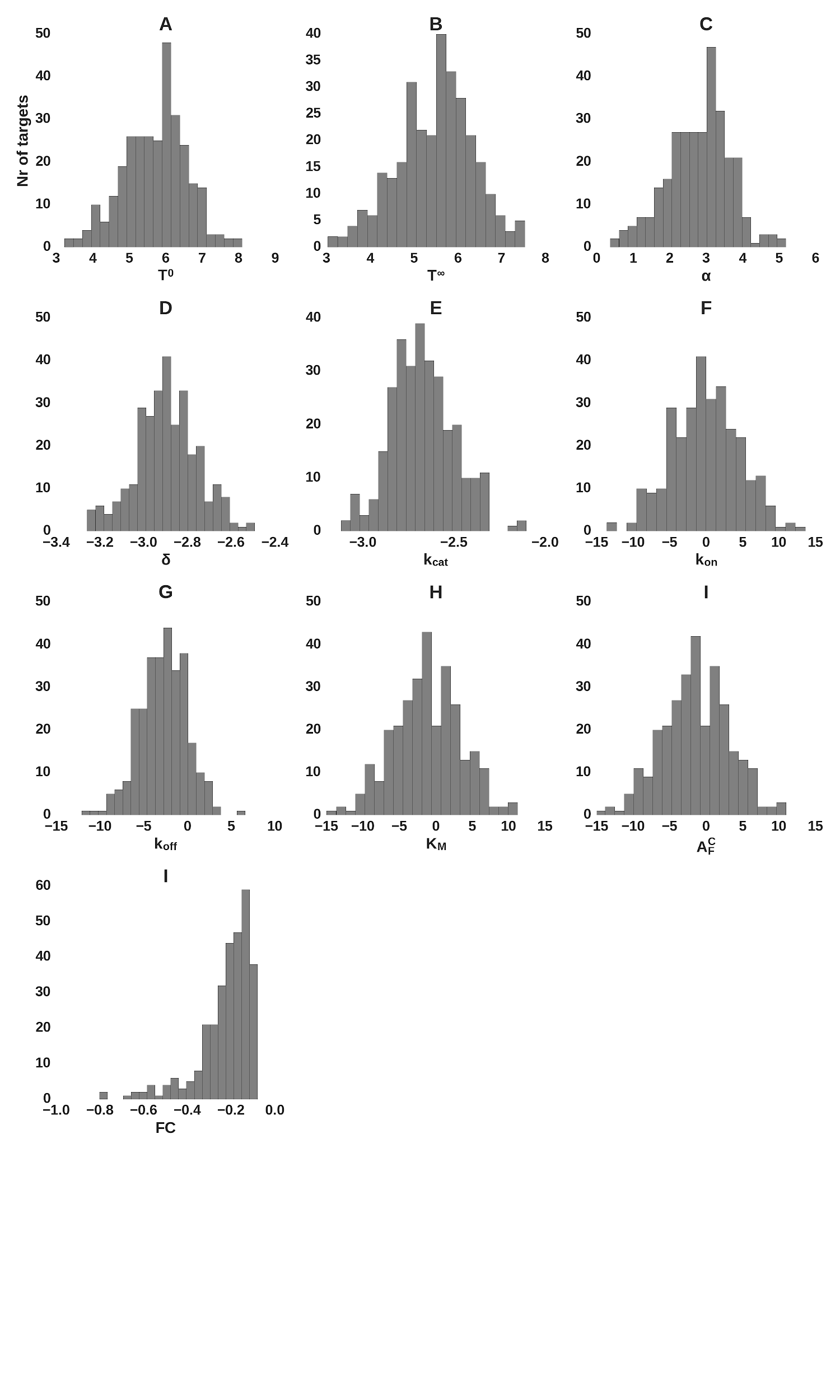


**Appendix Figure S4. Distribution of parameters of the *in silico* targets.** Log_2_ values are shown. See section ‘*In silico* analysis’ for additional explanation.


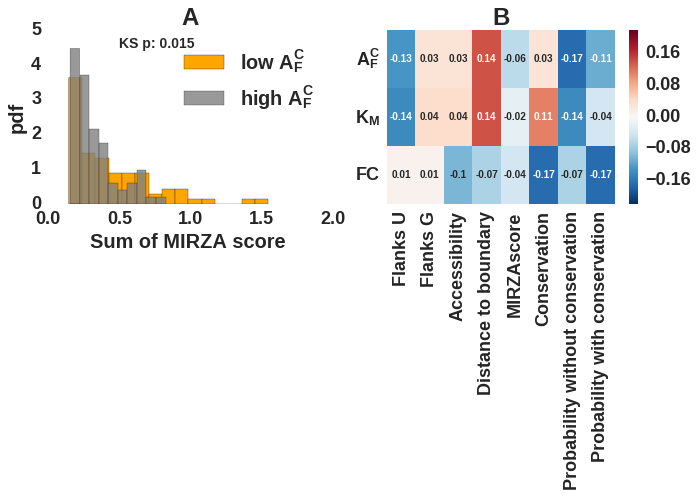


**Appendix Figure S5. hsa-miR-199a-3/5p targets** $A_{F}^{C}$ **correlate with binding site properties.**  **A**. Low $A_{F}^{C}$targets have higher MIRZA-G-C scores compared to high $A_{F}^{C}$targets. For each gene, we calculated the mean of the $A_{F}^{C}$values inferred from the i199 and i199-KTN1 data. We also calculated total MIRZA-G-C scores for each gene by summing the prediction scores for the two miRNAs. We took the union of the 40 targets with with lowest $A_{F}^{C}$for the two miRNAs (77 targets, as some were targeted by both miRNAs) and the similar list of targets with the highest $A_{F}^{C}$and compared their MIRZA-G-C scores. B. Spearman correlation of $A_{F}^{C}$, KM and FC to site properties. Binding site properties used to calculate MIRZA-G-C score for all targets of the two miRNAs which have only one binding site (of either of the two miRNAs, about 70% of targets) are used in this correlation. The partial properties are not additive and thus multi-site targets are omitted. Here ‘Probability with conservation’ is the MIRZA-G-C score.

##

##

##

#


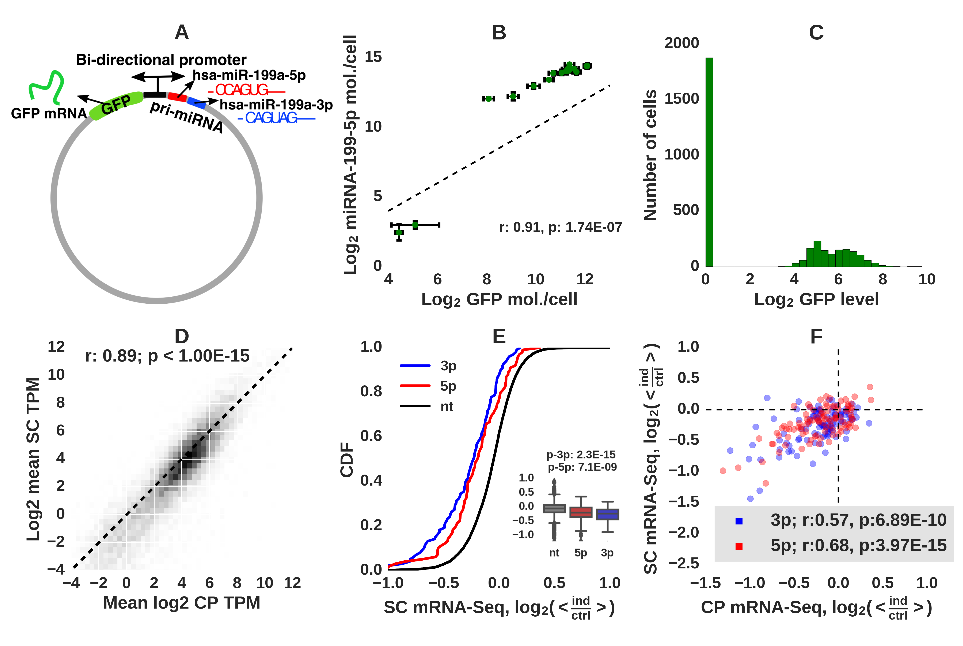


**Appendix Figure S6. Design and characterization of the experimental system. A.** Schematic representation of the construct used to express hsa-miR-199a-5p (red), hsa-miR-199a-3p (blue), and the reporter GFP mRNA from a bidirectional promoter. Shown are also the ‘seed’ sequences (nucleotides 2-7) of the two miRNAs. **B.** The expression levels of hsa-miR-199a-5p and GFP mRNA, measured from cell populations by quantitative PCR, are highly correlated. **C.** Histogram of normalized GFP mRNA expression (TPM) in individual i199 cells. **D.** Correlation of mRNA expression levels estimated from SC sequencing (1875 $T^{0}$cells (see text for definition) from which no GFP mRNA was captured) and from CP mRNA-seq (6 replicates of non-induced cell populations). **E.** Cumulative distribution of expression differences of the top 100 targets of hsa-miR-199a-5p (red), top 100 targets of hsa-miR-199a-3p (blue), and of 6179 remaining, ‘background’ genes (black) between cells expressing highest and lowest GFP levels (${216 T}^{\infty}$cells with > 6.8 TPM GFP (‘ind’) vs. 1875 $T^{0}$ cells with 0 TMP GFP (‘ctrl’)). Box plots of log2-fold change of non-targets, top 100 miR-199a-3p and top 100 miRNA-199a-5p targets are shown in the inset. P-values of the rank-sum test comparing targets and non-targets are also shown. **F.** Scatter plot of expression differences of the top 100 targets of each miRNA, estimated from bulk sequencing (CP) or from single cell sequencing ($T^{\infty}$and $T^{0}$cells defined as for previous panel). Similar to main Figure 1, but using TargetScan 6.2 instead of MIRZA-G-C-predicted targets.

#


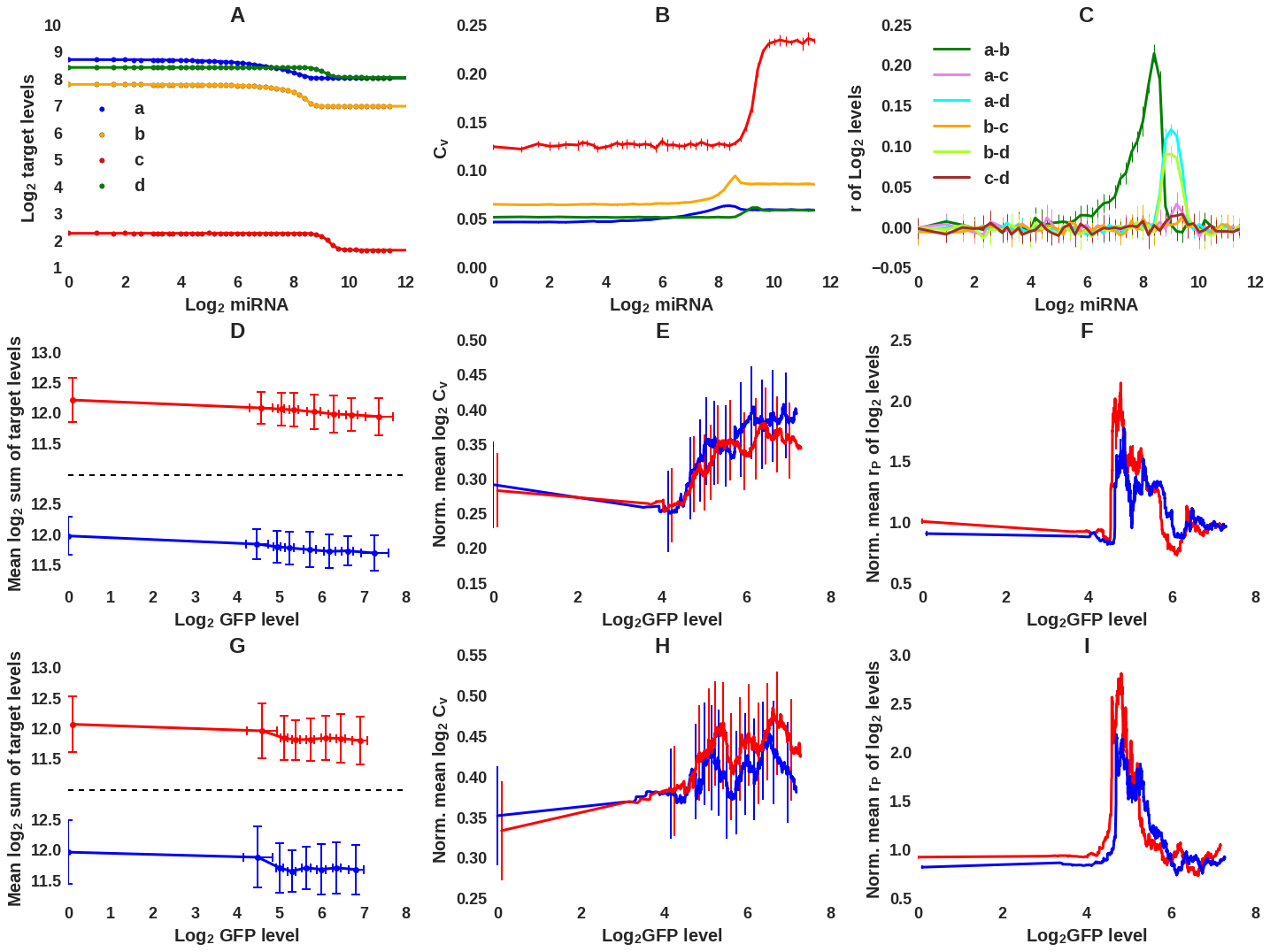


**Appendix Figure S7. Expected and observed response of miRNA targets to miRNA induction in single cells. A.** Results of numerical integration (Eqs (1), solid lines) and the average of six stochastic simulations (dots) of a model with four target genes (indicated by distinct colors) chosen to cover a wide expression range and to have either high or low sensitivity to the miRNA. Fifty *in silico* cells, each with a defined miRNA concentration were simulated. **B.** Coefficient of variation (C_V_) of *in silico* target levels across cells, calculated in function of miRNA expression, from the simulation trajectories. **C.** Pearson correlation coefficients of expression levels of pairs of genes from *in silico* cells, calculated in function of miRNA expression from the simulation trajectories. **D,G.** Total expression (log_2_ sum of TPMs) of 100 lowest $A_{F}^{C}$hsa-miR-199a-5p (red) and hsa-miR-199a-3p (blue) targets (see also Methods for target selection) in the i199 **(D)** and i199-KTN1 **(G)** cells, in function of log_2_ GFP expression in the same cells. **E,H.** Mean C_V_ and **F,I.** Mean Pearson pairwise correlation coefficients for miRNA targets in function of GFP expression in i199 **(E,F)** and i199-KTN1 **(H,I)** cells. Averages were calculated from the two hundred cells with GFP expression closest to a specific expression level. C_V_ values are shown as ratios to corresponding values computed for all mRNAs (**E,H)** and r_P_ to mean of 50 evaluations of random selection of 100 control genes (**F,I**). For **B,C,D** and **G** plot standard deviations are shown, for **E,F,H** and **I** standard error. Similar to main Figure 2, but using TargetScan 6.2 instead of MIRZA-G-C-predicted targets.

#


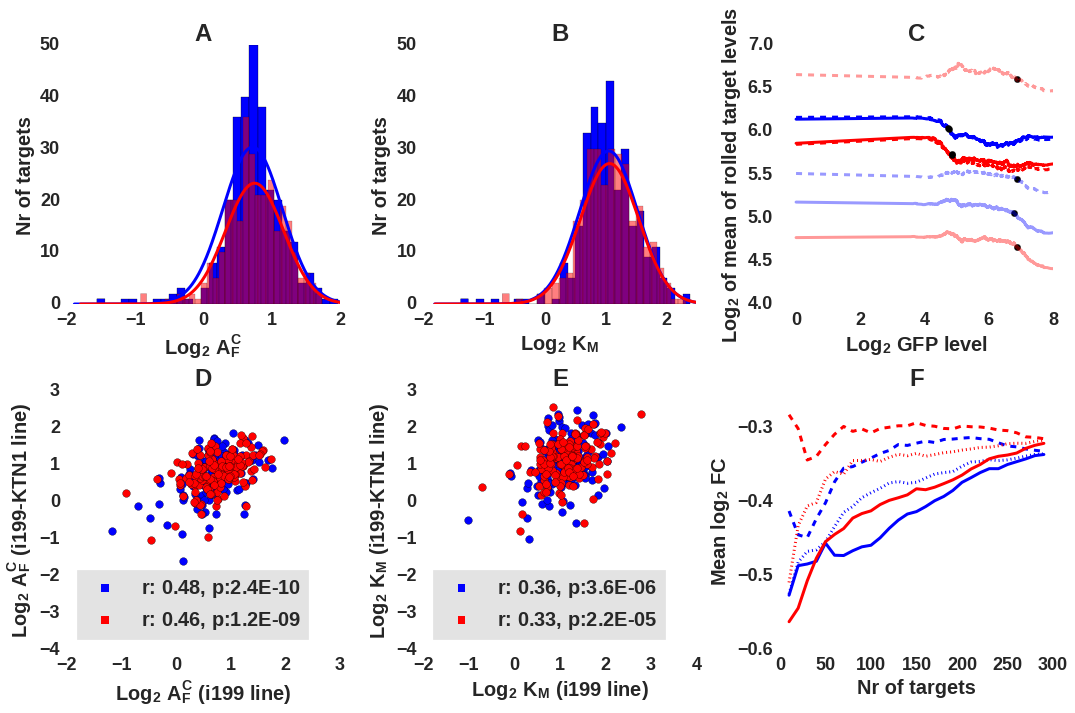


**Appendix Figure S8. Parameters describing the response of individual targets to changes in miRNA expression.** Histograms of $A_{F}^{C}$ **(A)** and *K_M_* **(B)** values of hsa-miR-199a-5p (red) and hsa-miR-199a-3p (blue) targets, inferred from the i199 cell line. The lines indicate the best-fitting Gaussian distributions. **C.** Response of hsa-miR-199a-5p (red) and hsa-miR-199a-3p (blue) targets to the miRNAs in i199 cells. Targets were selected based on $A_{F}^{C}$ (dashed lines) or *K_M_* (full lines) values, targets with low values of the respective parameters are shown in strong color and those with high values in faded colors. 20 targets were summed up for each category. Dots show the point where the targets have undergone ½ of their maximal down-regulation. **D.** Scatter plot of log_2_ $A_{F}^{C}$ values inferred for individual targets from the i199 and i199-KTN1 cell lines. Shown are also Pearson correlation coefficients and corresponding p-values. **E.** Scatter plot of log_2_ *K_M_* values inferred for individual targets from the i199 and i199-KTN1 cell lines. Shown are also Pearson correlation coefficients and corresponding p-values. **F.** Average log_2_ fold change of hsa-miR-199a-5p (red) and hsa-miR-199a-3p (blue) targets as a function of the number of top targets considered, where predictions are made based either on *K_M_* values (highest to lowest, full lines), $A_{F}^{C}$ values (lowest to highest, dashed lines) or Target-Scan context+scores scores (lowest to highest, dotted lines). Similar to main Figure 4, but using TargetScan 6.2 instead of MIRZA-G-C-predicted targets.

#


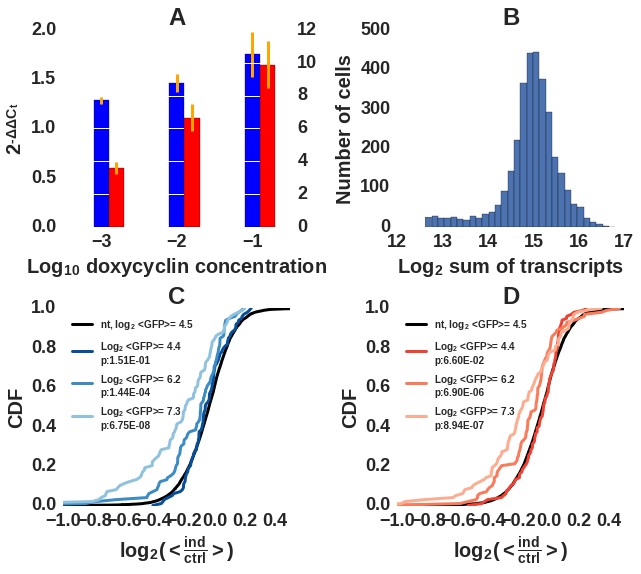


**Appendix Figure S9.** **Characterization of hsa-miR-199a-5p and hsa-miR-199a-3p miRNA activity. A.** Relative hsa-miR-199-3p (blue, left y-axis) and hsa-miR-199-5p (red, right y-axis) miRNA levels in doxycycline-induced cells compared to the non-induced cells, measured by quantitative PCR, demonstrate that the two miRNAs are co-expressed. The C_t_ values obtained for each set were normalized to the levels of hsa-miR-16 and to the values from non-induced cells. Error bars indicate standard deviations from 2 experiments. **B**. Expression of miRNAs in fully induced HEK cells as measured by Clip-Seq. Two replicates are correlated, hsa-miR-199-5p and hsa-miR-199-3p are indicated. **C.** Number of transcripts identified in individual i199 cells. **D,E.** Downregulation of top 100 predicted targets of the miRNAs in i199 cells with different levels of GFP. Three sets of cells (200 cells each) with increasing GFP expression levels were used, showing that the downregulation of hsa-miR-199-3p (blue lines, **D**), and hsa-miR-199-5p (red lines, **E**) targets increases with level of GFP expression; the distribution of log- fold changes of non targets is shown in black. *P* values are from the Kolmogorov-Smirnov test comparing the distributions of targets with that of non-targets. Similar to Figure S1, but using TargetScan 6.2 instead of MIRZA-G-C-predicted targets.

#


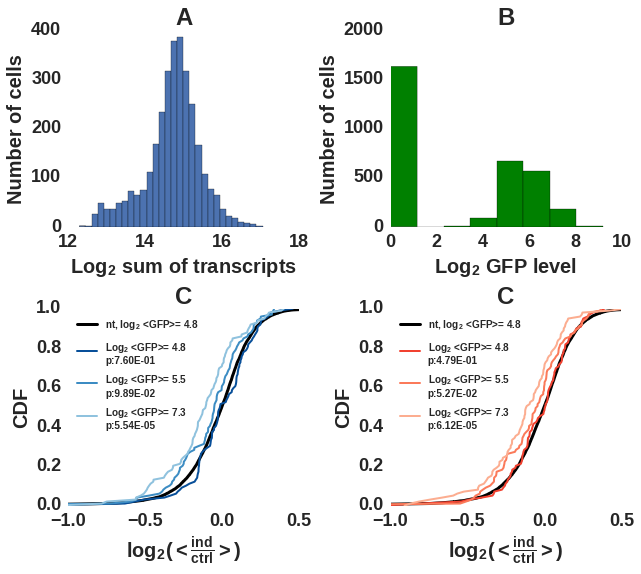


**Appendix Figure S10. Characterization of miRNA activity in single i199-KTN1 HEK cells**. **A.** Count of transcripts identified from each individual gene in single i199-KTN1 cells. **B.** Normalized GFP mRNA expression distribution in single cells. **C,D.** Downregulation of top 100 predicted targets of the miRNAs in i199-KTN1 cells with different levels of GFP. Three sets of cells (200 cells each) with increasing GFP expression levels were used, showing that the downregulation of hsa-miR-199-3p (blue lines, **C**), and hsa-miR-199-5p (red lines, **D**) targets increases with level of GFP expression; the distribution of log-fold changes of non targets is shown in black. Similar to Figure S2, but using TargetScan 6.2 instead of MIRZA-G-C-predicted targets.

#


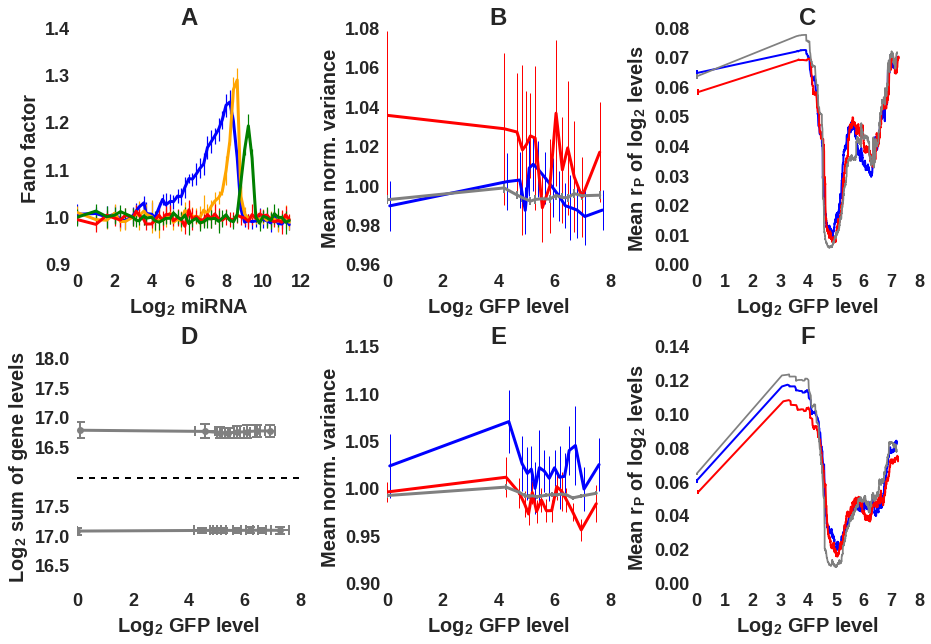


**Appendix Figure S11. Expected and observed response of miRNA targets to miRNA induction in single cells; additional information. A.** Fano factor of *in silico* target levels across cells, calculated in function of miRNA expression, from the simulation trajectories. The panel corresponds to panel B, Figure 2, where C_V_ is calculated using the same data. **B, E.** Normalized variance (using PAGODA package (Fan *et al*, 2016)) of 100 lowest $A_{F}^{C}$hsa-miR-199a-5p (red) and hsa-miR-199a-3p (blue) targets and all genes (grey) in the i199 **(B)** and i199-KTN1 **(E)** cells, in function of log_2_ GFP expression in the same cells; see Methods about PAGODA normalization and calculation details. **D.** Total expression (log_2_ sum of TPMs) of 1000 random genes in the i199 **(**lower line**)** and i199-KTN1 **(**upper line**)** cells. **C,F.** Mean Pearson pairwise correlation coefficients for miRNA targets in function of GFP expression in i199 **(C)** and i199-KTN1 **(F)** cells. Mean from 50 calculation evaluations of random selection of 100 genes is shown as grey line. Means were calculated from the two hundred cells with GFP expression closest to a specific expression level (**C,D,F**). For **A** and **D** panels standard deviations are shown, for **B,C,E** and **F** standard error. Similar to Figure S3, but using TargetScan 6.2 instead of MIRZA-G-C-predicted targets.

#


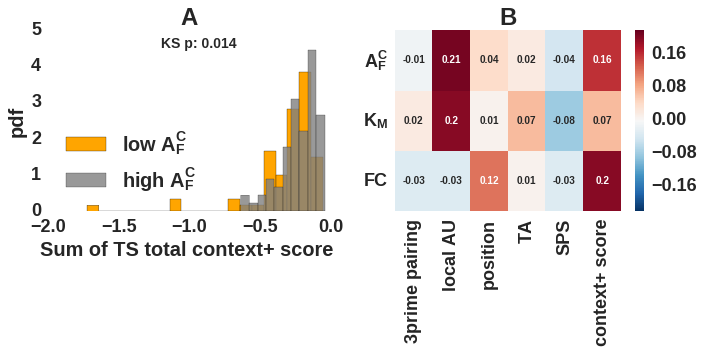


**Appendix Figure S12. hsa-miR-199a-3/5p targets** $A_{F}^{C}$ **correlate with binding site properties.**  **A**. Low $A_{F}^{C}$targets have lower Target-Scan scores compared to high $A_{F}^{C}$targets. For each gene, we calculated the mean of the $A_{F}^{C}$values inferred from the i199 and i199-KTN1 data. We also calculated total Target-Scan scores for each gene by summing the prediction scores for the two miRNAs. We took the union of the 40 targets with with lowest $A_{F}^{C}$for the two miRNAs (72 targets, as some were targeted by both miRNAs) and the similar list of targets with the highest $A_{F}^{C}$and compared their Target-Scan scores. **B.** Spearman correlation of $A_{F}^{C}$, K_M_ and FC to site properties. Binding site properties used to calculate Target-Scan context+score for all targets of the two miRNAs which have only one binding site (of either of the two miRNAs, about 70% of targets) are used in this correlation. Note that features that form Target-Scan and the context+score have minus values, as opposite to MIRZA-G-C score and its partial elements. Similar to Figure S5, but using TargetScan 6.2 instead of MIRZA-G-C-predicted targets.

##

##

##

##

**Appendix tables**

**Appendix Table S1. ‘Molecular function’ GO categories enriched in targets with low** $\boldsymbol{A}_{\boldsymbol{F}}^{\boldsymbol{C}}$. The union of the 40 targets with the lowest $A_{F}^{C}$for each of the two miRNAs (mean over i199 and i199-KTN1 data, 74 targets in total) was used as foreground set. As a background the joint list of targets (all for which $A_{F}^{C}$values were calculated) for 3p and 5p arm (present in both i199 and i199-KTN1 data sets; 301 in total) was used. 15 of the 74 targets are annotated with GO terms 2,3,5,10-12, related to transcription regulation and DNA binding.

| Nr | GO name | GOMFID | Pvalue | ExpCount | Count | Size |
| --- | --- | --- | --- | --- | --- | --- |
| 1 | hydrolase activity, acting on carbon-nitrogen (but not peptide) bonds | GO:0016810 | 0.00376701 | 1.0070922 | 4 | 4 |
| 2 | transcriptional activator activity, RNA polymerase II core promoter proximal region sequence-specific binding | GO:0001077 | 0.003860534 | 2.0141844 | 6 | 8 |
| 3 | RNA polymerase II transcription factor activity, sequence-specific DNA binding | GO:0000981 | 0.03697759 | 4.2801418 | 8 | 17 |
| 4 | syntaxin binding | GO:0019905 | 0.050522247 | 1.0070922 | 3 | 4 |
| 5 | histone deacetylase activity | GO:0004407 | 0.062719265 | 0.5035461 | 2 | 2 |
| 6 | monooxygenase activity | GO:0004497 | 0.062719265 | 0.5035461 | 2 | 2 |
| 7 | deacetylase activity | GO:0019213 | 0.062719265 | 0.5035461 | 2 | 2 |
| 8 | heme binding | GO:0020037 | 0.062719265 | 0.5035461 | 2 | 2 |
| 9 | tau protein binding | GO:0048156 | 0.062719265 | 0.5035461 | 2 | 2 |
| 10 | sequence-specific DNA binding | GO:0043565 | 0.065069175 | 6.2943262 | 10 | 25 |
| 11 | helicase activity | GO:0004386 | 0.069707031 | 1.7624113 | 4 | 7 |
| 12 | transcription regulatory region sequence-specific DNA binding | GO:0000976 | 0.097208599 | 5.035461 | 8 | 20 |

**Appendix Table S2. ‘Molecular function’ GO category analysis for high** $\boldsymbol{A}_{\boldsymbol{F}}^{\boldsymbol{C}}$**targets**. The union of the 40 targets with the highest $A_{F}^{C}$for each of the two miRNAs (mean over i199 and i199-KTN1 data, 77 targets in total) was used as foreground set. As a background the joint list of targets (all for which $A_{F}^{C}$values were calculated) for 3p and 5p arm (present in both 199 and i199-KTN1 data sets; 301 in total) was used.

| Nr | GO name | GOMFID | Pvalue | ExpCount | Count | Size |
| --- | --- | --- | --- | --- | --- | --- |
| 1 | organic anion transmembrane transporter activity | GO:0008514 | 0.01355234 | 0.7234043 | 3 | 3 |
| 2 | transmembrane transporter activity | GO:0022857 | 0.02635304 | 2.6524823 | 6 | 11 |
| 3 | transferase activity | GO:0016772 | 0.03671596 | 5.787234 | 10 | 24 |
| 4 | substrate-specific transporter activity | GO:0022892 | 0.04285169 | 3.6170213 | 7 | 15 |
| 5 | carbohydrate derivative binding | GO:0097367 | 0.05152047 | 11.0921986 | 16 | 46 |
| 6 | NAD+ kinase activity | GO:0003951 | 0.05749476 | 0.4822695 | 2 | 2 |
| 7 | diacylglycerol kinase activity | GO:0004143 | 0.05749476 | 0.4822695 | 2 | 2 |
| 8 | organic acid transmembrane transporter activity | GO:0005342 | 0.05749476 | 0.4822695 | 2 | 2 |
| 9 | amino acid transmembrane transporter activity | GO:0015171 | 0.05749476 | 0.4822695 | 2 | 2 |
| 10 | antiporter activity | GO:0015297 | 0.05749476 | 0.4822695 | 2 | 2 |
| 11 | adrenergic receptor binding | GO:0031690 | 0.05749476 | 0.4822695 | 2 | 2 |
| 12 | metal ion transmembrane transporter activity | GO:0046873 | 0.05749476 | 0.4822695 | 2 | 2 |
| 13 | catalytic activity | GO:0140103 | 0.05749476 | 0.4822695 | 2 | 2 |
| 14 | adenyl nucleotide binding | GO:0030554 | 0.07966644 | 9.8865248 | 14 | 41 |
| 15 | anion binding | GO:0043168 | 0.08369943 | 13.5035461 | 18 | 56 |
| 16 | purine ribonucleotide binding | GO:0032555 | 0.085241 | 10.8510638 | 15 | 45 |
| 17 | ion transmembrane transporter activity | GO:0015075 | 0.08612716 | 1.1732852 | 3 | 5 |
| 18 | ATP binding | GO:0005524 | 0.08957296 | 9.1631206 | 13 | 38 |
| 19 | calmodulin binding | GO:0005516 | 0.09252429 | 1.2056738 | 3 | 5 |
| 20 | cation transmembrane transporter activity | GO:0008324 | 0.09252429 | 1.2056738 | 3 | 5 |
| 21 | tubulin binding | GO:0015631 | 0.09660753 | 2.6524823 | 5 | 11 |

**Appendix References**

[Fan J, Salathia N, Liu R, Kaeser GE, Yung YC, Herman JL, Kaper F, Fan J-B, Zhang K, Chun J & Kharchenko PV (2016) Characterizing transcriptional heterogeneity through pathway and gene set overdispersion analysis. *Nat. Methods* **13:** 241–244](http://paperpile.com/b/tsJs0v/hUIF)
